# Introduction to a Twin Dual-Axis Robotic Platform for Studies of Lower Limb Biomechanics 

**DOI:** 10.1109/JTEHM.2023.3271446

**Published:** 2023-04-28

**Authors:** Joshua B. Russell, Connor M. Phillips, Matthew R. Auer, Vu Phan, Kwanghee Jo, Omik Save, Varun Nalam, Hyunglae Lee

**Affiliations:** School for Engineering of Matter, Transport and EnergyArizona State University 7864 Tempe AZ 85287 USA; School of Biomedical EngineeringNorth Carolina State University 6798 Raleigh NC 27695 USA

**Keywords:** Assistive robotics, medical robots and systems, rehabilitation robotics

## Abstract

This paper presents a twin dual-axis robotic platform system which is designed for the characterization of postural balance under various environmental conditions and quantification of bilateral ankle mechanics in 2 degrees-of-freedom (DOF) during standing and walking. Methods: Validation experiments were conducted to evaluate performance of the system: 1) to apply accurate position perturbations under different loading conditions; 2) to simulate a range of stiffness-defined mechanical environments; and 3) to reliably quantify the joint impedance of mechanical systems. In addition, several human experiments were performed to demonstrate the system’s applicability for various lower limb biomechanics studies. The first two experiments quantified postural balance on a compliance-controlled surface (passive perturbations) and under oscillatory perturbations with various frequencies and amplitudes (active perturbations). The second two experiments quantified bilateral ankle mechanics, specifically, ankle impedance in 2-DOF during standing and walking. The validation experiments showed high accuracy of the platform system to apply position perturbations, simulate a range of mechanical environments, and quantify the joint impedance. Results of the human experiments further demonstrated that the platform system is sensitive enough to detect differences in postural balance control under challenging environmental conditions as well as bilateral differences in 2-DOF ankle mechanics. This robotic platform system will allow us to better understand lower limb biomechanics during functional tasks, while also providing invaluable knowledge for the design and control of many robotic systems including robotic exoskeletons, prostheses and robot-assisted balance training programs. Clinical and Translational Impact Statement— Our robotic platform system serves as a tool to better understand the biomechanics of both healthy and neurologically impaired individuals and to develop assistive robotics and rehabilitation training programs using this information.

## I. Introduction

The ability to maintain postural balance is crucial for completing everyday tasks safely, and a reduction in fine postural balance control due to environmental perturbations, amputation, and/or neurodegenerative disorders can lead to severe impact in quality of life and a higher risk of falls [1], [2]. Investigating human postural balance will advance our understanding of falls and facilitate the development of assistive exoskeletons to mitigate the risk of falling and its consequences [3], [4] and rehabilitation robots for restoring balance control functions in people with disabilities [5], including stroke [6]. Recent works have also used information on human balance control to develop robot-assisted balance training for stroke patients [7], [8]. Hence, a detailed understanding of the mechanisms through which human postural balance is achieved and maintained, especially under various perturbations has tremendous applications for the improvement of human balance control and the development of assistive or rehabilitation robotics.

The human ankle plays a fundamental role in many lower extremity functions, including the manipulation of the body’s center of gravity during postural balance [9], [10], [11], and the transfer of energy during locomotion [12], [13]. These tasks are accomplished by the modulation of the ankle’s mechanical properties of stiffness and damping, also known as ankle impedance, via the human neuromuscular system. It is well-established that ankle impedance is dominated by ankle stiffness, and this is often the primary variable that is characterized in ankle impedance studies [14], [15], [16]. Detailed characterization of ankle impedance is key to understanding the contributions of the ankle during normal and altered postural balance control and locomotion. This information will allow us to better understand the effects of neuromuscular diseases on the lower extremity and develop therapies or assistive robotic devices [17], [18] to restore its function. Thus, it is imperative that high fidelity mechanical systems are produced to accurately measure and characterize ankle impedance.

Several robotic platforms capable of simulating challenging environmental conditions have been developed for characterizing human postural balance. The commercially available Biodex platform is the most widespread and has been used to investigate postural balance under tilted or shaking environments [19], [20]. Moreover, several research groups have developed their own customized platforms for specific purposes. One such group developed an electro-hydraulic based platform to generate oscillatory perturbations on the ankles and assessed postural balance during those perturbations while standing [21]. However, this platform is limited to single-axis perturbations, i.e., movement only in the dorsiflexion-plantarflexion (DP) or inversion-eversion (IE) at one time. Another specialized platform was designed with a parallel mechanical mechanism to produce perturbations simultaneously in both DP and IE directions for postural balance assessment [22]. Both systems possess a single force plate which limits analysis to the net center-of-pressure (CoP) displacement. The ability to assess bilateral CoP displacement is very important since the postural control between the left and right foot is often asymmetrical, even in healthy subjects, and the asymmetry can be more pronounced in people with balance disorders or other neuromuscular issues of the lower limbs [14].

There are also several systems developed to characterize ankle impedance, one in the form of a wearable ankle robot and two platform-based systems. The wearable ankle robot, dubbed the “Anklebot”, allowed for characterization of ankle impedance in multiple movement directions (DP and IE) through the swing, early stance, and toe off portions of the gait cycle. However, it was unable to characterize impedance during high load conditions at the ankle such as quiet standing or the mid/terminal stances of the gait cycle [23]. This shortcoming was addressed by two robotic platform devices. The position-controlled “Perturbator” robot described in [24] used slow ramp perturbations to characterize ankle impedance during the stance phase but was limited to characterization only in the DP direction, while the system described in [25] uses a torque-controlled vibrating robotic platform to characterize impedance in both the IE and DP directions with perturbations of less than 2° in amplitude. These robotic platforms possess several limitations that prevent comprehensive analysis of ankle impedance. Both systems possess only a single mode of operation which prevents them from simulating real-world environments (e.g., compliant surfaces) that are encountered in daily life. In addition, the limited range of motion of these systems does not allow them to capture the dynamics of ankle impedance during postural balance in standing.

Previously, we developed a 2 degree-of-freedom (DOF), impedance-controlled, robotic platform that addressed the limitations of other existing platform-based systems. This device was capable of measuring ankle impedance in both the IE and DP directions during standing and walking, possessed multiple control methods, and had a much greater range of motion (±15° in DP and ±10° in IE) [26]. It also demonstrated the ability to improve paretic ankle control in patients with stroke [7]. However, this device itself was limited to measurements of one-side (right leg) ankle impedance [15] or postural balance studies where both feet were placed side-by-side on the single platform [27], [28].

The motivation of this paper is to develop a system that overcomes the limitations of our previous work and other state-of-the-art balance systems. The system presented here can measure postural balance while simulating various environmental conditions and can also measure bilateral, 2-DOF, ankle impedance during both standing and walking. We designed several human experiments to demonstrate the effectiveness of this system in evaluating postural balance and ankle mechanics under these unique conditions. The four we include here represent a diverse sample of the possible experiments. The first two experiments will demonstrate the ability of the system to measure postural balance while simulating challenging environments including a compliant impedance-controlled setting with various stiffnesses and oscillatory perturbations with varying amplitudes and frequencies. These experiments were designed to emulate the challenging environmental perturbations that an individual may be exposed to during daily activities. The term “environmental perturbation” refers to balance threats that are imposed by the environment in which an individual is standing. For example, standing on coarse sand (i.e., compliant surface) or transportation on vehicles (i.e., oscillatory surface). The compliant surface experiment employs a passive perturbation where the response of the user creates the balance threat. This is opposed to the oscillatory perturbation which is an active perturbation and remains constant regardless of user behavior. These experiments are meant to represent a sample of the novel environmental conditions which can be simulated with our device. The final two experiments will demonstrate the system’s ability to measure bilateral ankle impedance in 2-DOF during standing and walking. This robotic platform system will not only allow us to better understand human biomechanics and motor control but also provide invaluable knowledge for the design and control of many robotic systems including robotic exoskeletons, prostheses, or robot-assisted balance training programs.

## II. Twin Dual-Axis Robotic Platform

### A. Design of the Twin Dual-Axis Robotic Platform

The robotic platform may be applied to study a wide range of lower limb biomechanical factors. To support these numerous potential applications and address the limitations of previous work, the system was designed to satisfy several performance criteria. Each of the platforms were designed to rotate in both the IE and DP directions. Furthermore, each DOF was designed to have an appropriate range of motion to accommodate the full range of motion of the human ankle, ±15° and ±10° from the horizontal plane for DP and IE, respectively [29].

In addition, the platform was designed to simulate a wide range of mechanical environments using an admittance controller which can emulate highly compliant to highly stiff surfaces. They were also designed with the ability to apply rotational perturbations up to 100°/s to ensure that the intrinsic, reflexive, and voluntary ankle mechanics can be reliably characterized [30]. Lastly the platform was designed to support a subject of up to 100 kg during dynamic conditions since this weight limit covers 80% of the population [31]. This demands a maximum motor torque of 455 Nm and 195 Nm for DP and IE, respectively [26].

**FIGURE 1.**
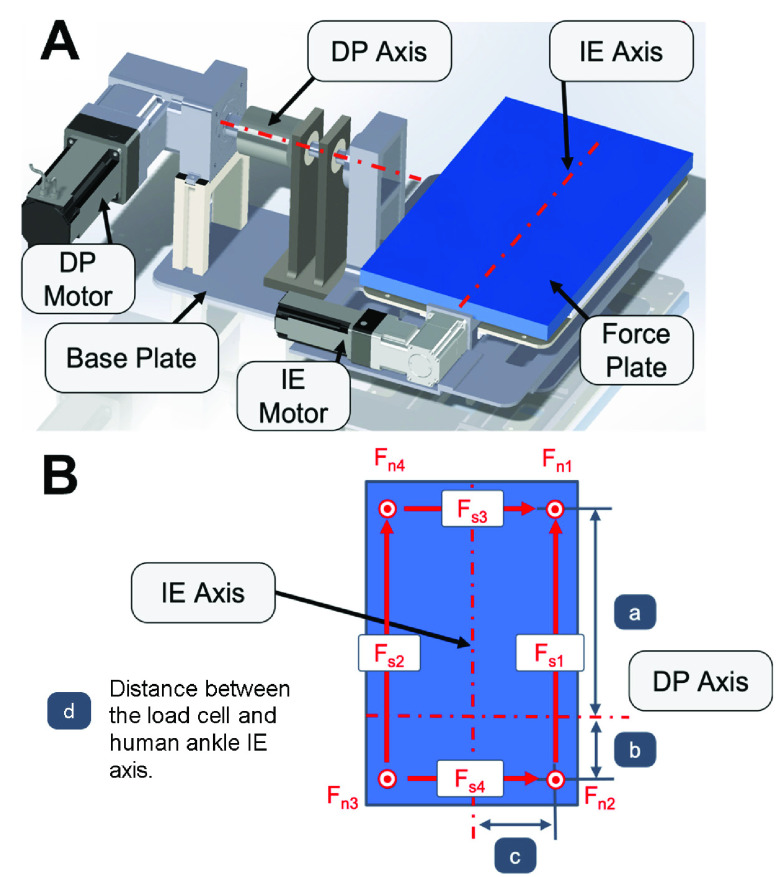
(A) Mechanical components and critical axes of the twin-axis platform. (B) Dimensions used in joint torque calculations.

The independent axes of each platform are designed with two motors perpendicular to each other that rotate their respective platforms. The IE subassembly involving the IE motor, gearbox, IE plate and the force plate (9260AA3, Kistler, New York, USA) are mounted on top of the DP moving platform (Fig. 1 A). The DP plate is supported via rollers on the support plate and rotated with the DP motor fixed to the support plate. The DP motor is considerably more powerful than the IE motor because it will rotate the IE subassembly and requires more torque due to the larger moment arm about its rotational axis. With the combination of these two subassemblies, the force plate can rotate about two perpendicular axes. The IE motor axis is below the human ankle IE axis to maintain functionality of the system without introducing unnecessary complexity in the case of a Stewart Platform and other remote center of rotation mechanisms [32]. This configuration of the IE axis causes the ankle to translate slightly when rotated, however, a pilot study conducted with the first prototype of the twin-axis robotic platform showed the effect to be negligible [26].

### B. Data Acquisition and Processing

Accurate kinematic information is required to characterize mechanical properties of the human ankle. The angles of the platform axes are recorded based off the 16-bit encoder attached to the servomotors. The data acquisition (DAQ) unit resolves analog voltages with 16-bit resolution at a rate of 2 kHz. Angular data is filtered using a $4^{th}$ order butterworth low-pass filter with a cutoff frequency of 15 Hz [33]. Any higher order kinematic data is calculated using a five-point midpoint numerical derivative.

The torque at the ankle joint is also required to characterize mechanical properties of the ankle. Each force plate has 8 channels of force measurement data. They measure the normal force in each of the 4 corners as well as the shear force along each edge (Fig. 1 B). The force plate data was recorded at a sampling rate of 2 kHz, and was low pass filtered using a $4^{th}$ order butterworth filter with a cut-off frequency of 15 Hz [34]. The torques about the ankle are calculated using these 8 signals as in eq. (1) and eq. (2) [26]:

\begin{align*} \tau _{Ankle_{D}P }&=\left ({F_{n 1}+F_{n 4}}\right) a-\left ({F_{n 2}+F_{n 3}}\right) b+\left ({F_{s 1}+F_{s 2}}\right) d \\ &\quad - \tau _{Platform\_{}D P} \tag{1}\\ \tau _{Ankle_{I}E }&=\left ({F_{n 1}+F_{n 2}-F_{n 3}-F_{n 4}}\right) c+\left ({F_{s 3}+F_{s 4}}\right) d \\ &\quad - \tau _{Platform\_{}IE} \tag{2}\end{align*}

where $\tau _{Ankle\_{}DP}$ and $\tau _{Ankle\_{}IE}$ refer to the torque about the human ankle DP and IE axes, respectively. $\text{F}_{n1}$ through $\text{F}_{n4}$ correspond to the normal force measured at each corner, and $\text{F}_{s1}$ through $\text{F}_{s4}$ correspond to the shear force between the sides of the four corners which are computed in the force plate signal processor. The dimensions $a$ through $c$ represent the location of the load cells to the rotational axes of the platforms while dimension $d$ is the height distance from the load cell to the IE axis of the average human ankle (Fig. 1 B). Variability in distance $d$ was shown to have an insignificant effect on ankle stiffness measurements in a previous study of 40 subjects [14]. Lastly, $\tau _{Platform\_{}DP}$ and $\tau _{Platform\_{}IE}$ are the measured torque induced by the motion of the platform in the DP and IE axes, respectively. These are determined from the platform system identification discussed in Section II-D and are subtracted from the total torque to account for the inertial effects of the moving platform.

The platform controllers are real-time target machines (Speedgoat, NATICK, MA, USA) using simulink real-time (Mathworks, NATICK, MA, USA). Each servo motor has A dedicated servo driver with a tuned velocity controller implemented (AKD-01206 AND AKD-00606 for DP and IE actuations respectively, Kollmorgen, Radford, VA). The tuning of the servo motor is done through the kollmorgen workbench software using system identification [35].

### C. System Integration

One aspect that needed to be considered after both platforms were assembled side-by-side was the coplanarity of the top surface. Care was taken to align the two platform surfaces because of the effect that surface misalignment would have on the weight distribution of subjects. The lateral distance between the two platforms was determined to ensure, in a worst-case condition, that clearance between the moving components is maintained and secondly, that the distance between the left and right IE axes is comfortable for the average subject. The platforms were placed as close as practically possible to minimize any impact the distance would have on postural stability. For any given subject, increasing stance width will improve stability and reduce fall incidents [36] and increased lateral stability would require much more aggressive postural stability perturbations to produce the same effect.

**FIGURE 2.**
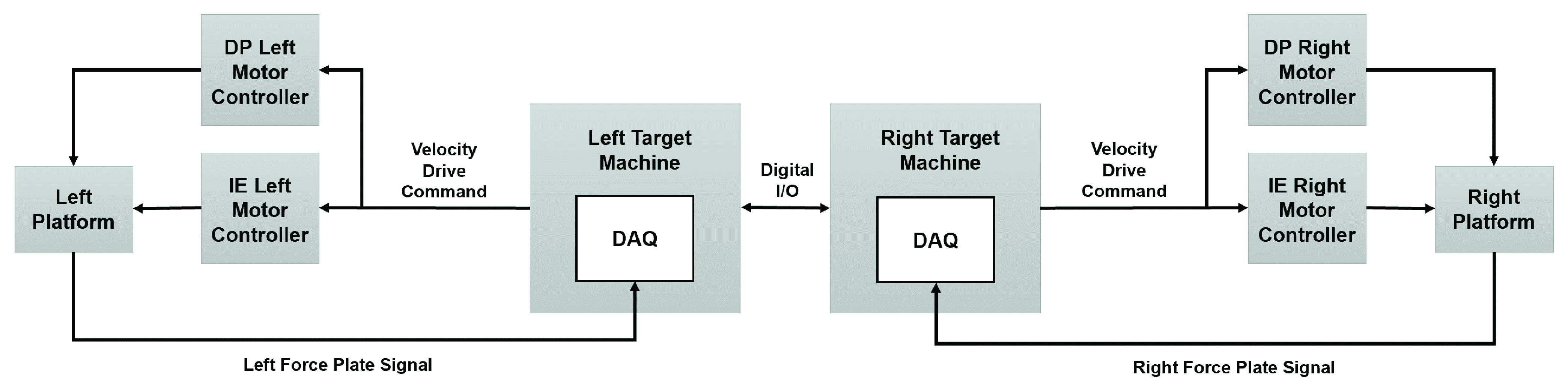
A system diagram for the twin dual-axis platform. See [26] for a detailed description of the motor controller and platform control scheme.

Another aspect considered was the synchronization between the two platforms. One real-time target machine set a designated digital I/O pin high, while the other real-time target machine had a designated input pin waiting to receive the signal (Fig. 2). Both signals were recorded from their respective units and the rising edge was used as the synchronization point during analyses.

### D. System Validation

Position control is used for many applications of the platform. For example, slower sinusoidal oscillations are used for postural balance while quick ramp-and-hold perturbations are used to characterize ankle impedance. The sinusoidal oscillations were tested in a range of 0.5 to 1.5 Hz with an amplitude of 8°. The accuracy of the position control for sinusoidal perturbations was measured using a motion capture system (Flex 3, OptiTrack, Corvallis, OR, USA).

The mean absolute difference in angle from the expected sinusoidal trajectory to the motion capture data was 0.049° and 0.025° for the left and right [26] platforms, respectively. Ramp-and-hold perturbations were tested up to 100 °/s but reported at 45°/s since this is the maximum speed used during ankle impedance characterization. The accuracy of ramp and hold perturbations at 45°/s was measured using the phase difference and steady-state error for no loading, static loading (under human weight during standing), and dynamic loading (during walking) conditions for each motor (Table 1) as compared to an ideal 3° ramp-and-hold perturbation with minimum jerk trajectory. The perturbations were aligned at 0.01° and the phase difference was calculated as the mean difference in time from 0.25° to 2.75°. The range 0.25° to 2.75° was chosen to prevent potential contributions from the steady-state error. Steady-state error was calculated as the mean absolute difference in angle of the ideal perturbation and each condition between 0.15 s to 0.25 s which was 0.025 s after the end of the ideal perturbation. These errors were minimal and probably accumulated from the backlash. The maximum allowable backlash for all gearboxes is 0.083° [37].

**TABLE 1:** Ramp and Hold Perturbation Accuracy Under Various Loading Conditions

	LEFT				RIGHT			
	DP		IE		DP		IE	
Testing Condition	Phase (ms)	SS_err_(deg)	Phase (ms)	SS_err_(deg)	Phase (ms)	SS_err_(deg)	Phase (ms)	SS_err_(deg)
No loading	0.19	0.037	0.20	0.100	0.37	0.036	0.36	0.108
Static loading	0.18	0.038	0.31	0.088	0.12	0.037	0.52	0.101
Dynamic loading	0.12	0.074	1.69	0.187	0.71	0.048	0.47	0.100

The platform dynamics also need to be characterized so they can be removed from the system when characterizing the human ankle. Ramp-and-hold perturbations with an amplitude of 3° and speed of 45°/s were used to determine the dynamic torques with known kinematic data. The mechanical impedance parameters, i.e., stiffness, damping and inertia can be determined using a $2^{nd}$ order system regression analysis. The system is very rigid which results in inertia being the dominant factor contributing to the system dynamics. Dynamics of both left and right platforms are more than 99% dominated by inertia. Thus, stiffness and damping contributions to the platform dynamics torque can be considered negligible.

The platform must also be able to simulate various mechanical impedances or variable stiffness, damping and inertia values [38], [39]. The most important property to validate is stiffness to allow for postural balance studies to be conducted on these platforms. In these studies, stiffness is the property modulated to simulate varying mechanical environments. This was done by placing known weights on the platforms to cause rotational displacements given a known expected, simulated stiffness [26]. We chose to present only stiffness validation here because accurate simulation of this parameter is essential to our human experiment on compliant balancing surfaces. The stiffness range was validated from very compliant, 50 Nm/rad, to very stiff, 2000 Nm/rad (Table 2). The actual stiffness was computed using the measured torque divided by the measured displacement. The maximum error in simulated stiffness was 4.3% percent in the 2000 Nm/rad condition, which is an inconsequential amount for the intended use cases of the twin-axis dual platform system.

**TABLE 2:** Comparison of Commanded And Estimated Stiffness Of The Platforms

	LEFT				RIGHT [26]			
Commanded Stiffness (Nm/rad)	Est. DP Stiffness (Nm/rad)	Error (%)	Est. IE Stiffness (Nm/rad)	Error (%)	Est. DP Stiffness (Nm/rad)	Error (%)	Est. IE Stiffness (Nm/rad)	Error (%)
50	49.2	1.69%	50.3	0.52%	49.5	0.92%	49.0	2.04%
250	254.1	1.61%	259.7	3.72%	254.5	1.81%	248.9	0.45%
500	507.7	1.52%	513.4	2.99%	505.7	1.13%	494.1	1.18%
1000	1036.2	3.49%	1040.3	3.87%	1007.5	0.75%	1011.0	1.10%
2000	2089.9	4.30%	2086.3	4.14%	1995.2	0.21%	2014.7	0.74%

The platforms must also be able to accurately measure the impedance of the human ankle. This was verified by characterizing the mechanical properties of a mock ankle device, a method previously implemented in [26]. The mean stiffness value for the IE direction was 50.75 Nm/rad with errors of 1.74 and 0.62 Nm/rad for right and left sides, respectively, while the mean stiffness value for the DP direction was 144.77 Nm/rad with errors of 3.64 and 5.94 Nm/rad for right and left sides, respectively. These values are much smaller than the typical within-subject variability of ankle stiffness [14].

## III. Human Experiments

The following sections will detail the human experiments performed to demonstrate the functionality of the twin dual-axis platform system. All subjects who participated in these experiments were free from any biomechanical injuries or neurological diseases that would affect their ability to maintain upright standing posture. The methods of this study were approved by the Institutional Review Board of Arizona State University (STUDY00012542 for the standing postural balance study and STUDY00012606 for the ankle impedance quantification study). Informed consent was provided by all subjects prior to their participation.

**FIGURE 3.**
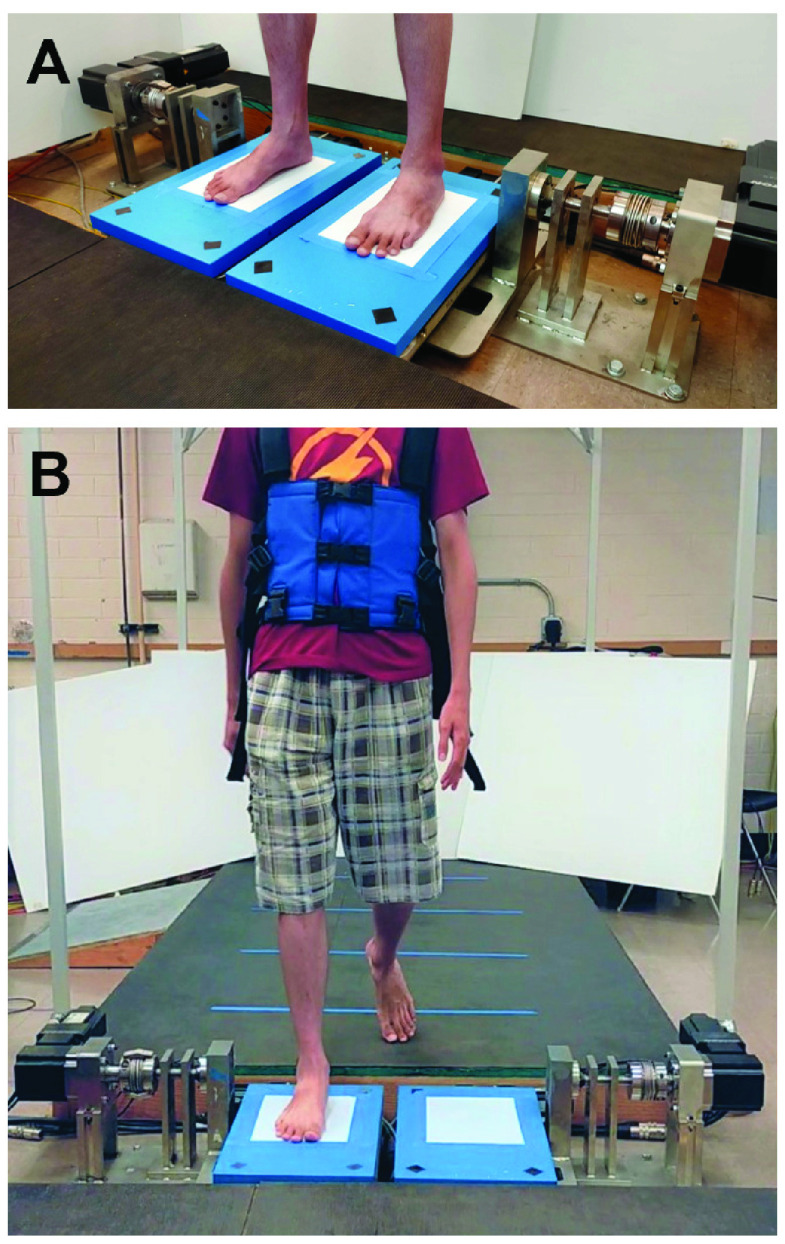
A subject on the platform during (A) standing and (B) walking experiments.

All subjects were requested to stand or step on each platform with their ankles in-line with the DP motor axes, and as near the lateral midline as possible without altering their normal foot posture (Fig. 3 A). All subjects wore a harness during experimentation which would prevent them from falling, but it was adjusted to have no weight support which could potentially affect their natural weight distribution.

### A. Standing Postural Balance Experiments

Five young healthy subjects (age: 24.6±3.5 years, height: 180.9±10.3 cm, and weight: 71.1±12.8 kg) were recruited to participate in two different experiments, one in which the system simulated various compliant environments (passive perturbations) and another with simulated oscillatory environments (active perturbations).

While maintaining upright standing posture on the platform, the subjects were exposed to different challenging environments simulated by the platform (details of each are presented in the next sub-section). At the same time, two force plates on top of the platform record center-of-pressure (CoP) displacement under each foot.

Subjects were instructed to minimize their arm motion and keep eyes straight towards a marker on a wall during the experiments. This marker assisted the subject in maintaining proper body orientation during the experiment.

#### 1) Postural Balance Under Passive Perturbations

The objective of this experiment was to assess human postural stability while standing on a range of compliant environments that passively perturbs human balance. The control variable was the ground stiffness simulated by the admittance controller of the platforms.

In this experiment, three compliant levels were chosen including rigid, compliant, and highly compliant corresponding to three simulated stiffness values of 10,000, 500, and 250 Nm/rad, respectively. These values were chosen based on our previous work with the single dual-axis robotic platform [26]. The same stiffness was simulated in both platforms for each compliant condition.

Three trials each lasting 60 seconds were performed for each compliant condition, summing to 9 trials in total. The order of trials was randomized for each subject. A 2-minute break was given if requested by the subject.

#### 2) Postural Balance Under Active Perturbations

Unlike the previous experiment which simulated a compliant environment that passively perturbed human balance, this experiment focused on evaluating the effects of oscillatory (specifically sinusoidal) perturbations on postural balance control. Therefore, the experiment contained two control variables including the amplitude and frequency of the input sinusoidal signal to the platforms.

In this preliminary study, we examined the effects of oscillatory perturbations created by combining three different amplitudes (0.5, 1.0, and 1.5°) with three different frequencies (0.5, 1.0, and 1.5 Hz). Like the previous experiment, both platforms possessed the same operating parameters for each experimental condition but the phase difference of 90° in the sinusoid signals between the left and right platforms was maintained so that the two platforms were not moving in the same direction.

There were three trials (each lasting 60 seconds) per condition for each of the 9 combinations of experimental variables for a total of 27 trials for each subject. The order of trials was randomized for each subject. A 2-minute break was given per subjects’ request or after every 9 trials.

#### 3) Data Analyses for Postural Balance Experiments

The net CoP displacements were calculated from CoP displacements under the right (*CoP* $_{right}$) and left (*CoP* $_{left}$) feet, using Eq. (3) [14]:

\begin{align*} {CoP}_{net}&=\left ({\frac {V R_{right }}{V R_{right }+V R_{left }}}\right) {CoP}_{right} \\ &\quad +\,\left ({\frac {V R_{left}}{V R_{right }+V R_{left}}}\right) {CoP}_{left} \tag{3}\end{align*}

where *VR* $_{right}$ and *VR* $_{left}$ stands for the vertical reaction forces measured from the right and left surfaces of the platform.

After the net CoP displacements were obtained, the total excursion (TOTEX) of the net CoP was calculated to quantify the stability of the subjects’ postural balance. An increase in the TOTEX outcome is related to the increase in postural sway which, in turn, indicates the decrease of postural stability during quiet standing. The calculation of this outcome was adopted from [19], as shown in Eq. (4):

\begin{align*} T O T E X&=\sum _{i=1}^{N}[\left ({{CoP}_{A P}[i+1]-{CoP}_{A P}[i]}\right)^{2} \\ &\qquad \qquad +\left ({{CoP}_{M L}[i+1]-{CoP}_{M L}[i]}\right)^{2}]^{\frac {1}{2}} \tag{4}\end{align*}

where $N$ is the total number of instantaneous points in the CoP series, *CoP* $_{AP}$ and *CoP* $_{ML}$ is the (net) CoP displacement in the anterior-posterior (AP) and medio-lateral (ML) directions, respectively.

#### 4) Results of Standing Balance Experiments

**FIGURE 4.**
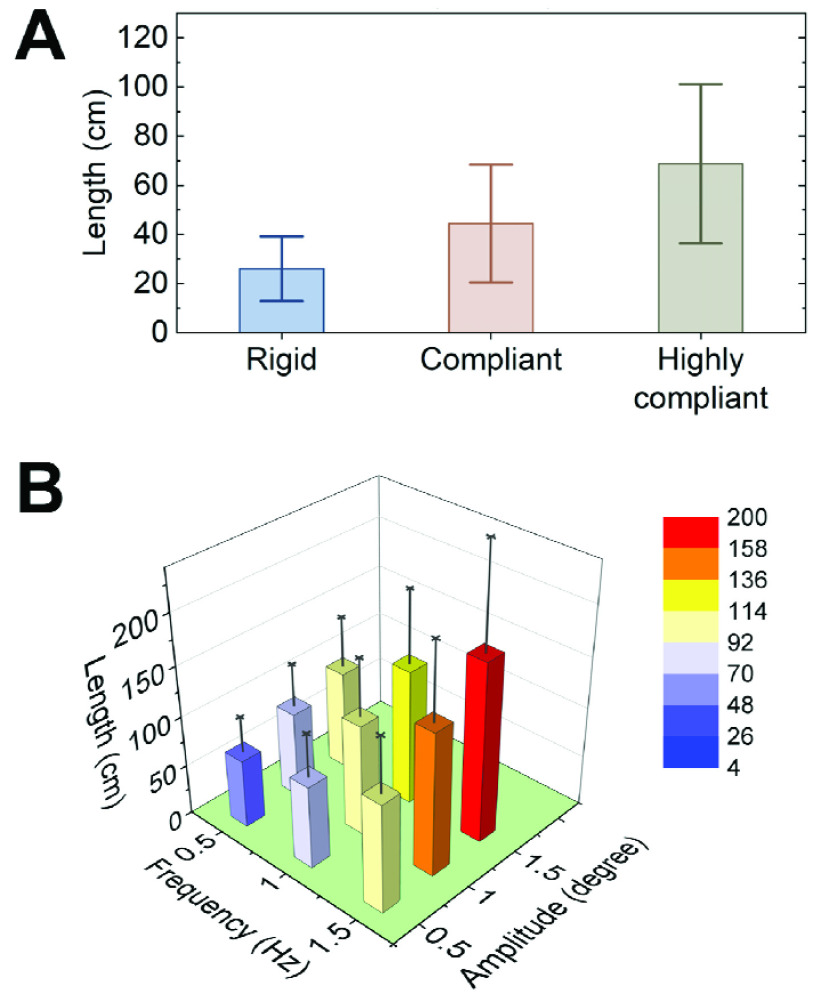
Results of the standing balance experiments. (A) Postural balance under (A) passive perturbations and (B) active perturbations). Average results (mean and mean ± 1 standard deviation) across all five subjects are shown.

Effects of compliant environments on postural balance can be seen in Fig. 4 A. The TOTEX averaged across subjects drastically increased from 26.0±12.7 cm during the rigid condition to 68.8±31.3 cm during the highly compliant condition. These observations revealed that increasing the compliance of the standing environment may negatively impact standing postural balance.

In addition, effects of oscillatory environments on standing postural balance can be seen in Fig. 4 B. Results showed that standing postural balance may also be negatively affected by increasing the oscillatory amplitude and/or frequency of the input sinusoidal signal, and the interaction between increasing amplitude and frequency may further worsen performance. This is evidenced by the increase of the averaged TOTEX from 68.2±35.6 cm during the minimum oscillatory condition (i.e., 0.5 Hz and 0.5°) to 178.2±100.5 cm during the maximum one (i.e., 1.5 Hz and 1.5°).

No statistical tests were performed due to the small sample size since this is beyond the scope of this work. However, the consistency in our preliminary results demonstrated that our system has a high potential to detect differences in balance while simulating increasingly challenging environments. Therefore, we expect statistically significant results with a larger sample size.

### B. Ankle Impedance Quantification Experiments

Five young healthy subjects (age: 22.7±4.2 years, height: 175.6±11.9 cm, weight: 70.7±12.0 kg) were recruited to take part in the data collection for quantifying the bilateral ankle impedance in the IE and DP directions during both standing and walking with the goal of assessing the level of symmetry between the dominant and non-dominant ankles. The walking IE direction data for one subject was removed, due to inability to place the foot correctly on the platform during experimentation. All subjects were right-side dominant.

The protocol for both impedance experiments involved a series of perturbations applied to the ankles of each subject. Each perturbation was 3° in magnitude, applied over 125 ms, with a peak velocity of 45°/sec and followed a minimum jerk velocity profile. Perturbations in the DP direction caused the foot to dorsiflex, while perturbations in the IE direction caused the foot to evert. Subjects also had a dual-axis goniometer (SG110, Biometrics Ltd, UK) placed on their ankle to record its angle of deflection.

#### 1) Ankle Impedance Quantification During Standing

The objectives of the standing impedance experiment were to evaluate the capability of the twin-axis dual platform system to accurately quantify ankle impedance in both legs during normal quiet standing and to assess the differences between the stiffnesses of the non-dominant and dominant ankles in both the DP and IE directions.

In this experiment, one trial consisted of a single perturbation to either the non-dominant or dominant ankle. Trials were grouped by the direction in which perturbations occurred (DP or IE). Kinematic data of the ankle and platforms as well as force data from each force plate were collected over 2 blocks with each block containing 15 trials for a total of 30 trials per foot per direction. Before the start of each set of blocks in the DP and IE directions, the subject’s nominal quiet standing CoP of each leg was found in the local coordinate frame of the corresponding platform. For the impedance characterization perturbations to occur, a set of conditions needed to be maintained for 0.5 s consecutively. The perturbations were only applied if: 1) the side-specific CoP remained within a 0.5 cm radius of the nominal CoP and 2) if the weight on that platform was within ±2 kg of half the subject’s bodyweight. A visual display was placed in front of the subject that indicated the magnitude of the weight on the foot under test and the respective platform’s local CoP. A random delay ranging from 0 to 0.5 s was included before the start of each perturbation to prevent the subject from predicting the perturbation occurrence.

#### 2) Ankle Impedance Quantification During Walking

The goals of the walking impedance experiment were to showcase the capability of the dual platform system to accurately quantify ankle impedance in the DP and IE directions for both non-dominant and dominant legs during the stance phase of walking and to assess the differences between the stiffnesses of the dominant and non-dominant ankles in both the DP and IE directions.

Subjects donned a safety harness and walked across an instrumented walkway leading to the platform (Fig. 3 B). Visual guides were placed on the walkway to encourage proper foot placement. A motion capture marker was placed on the subject’s foot to indicate the location of the ankle and its axis of rotation. Subjects were instructed to walk in sync with a metronome operating at 100 beats-per-minute. Perturbations were set to occur at $\sim $45% of the stance phase. In this experiment, a trial consisted of one step onto the platform. Half of the trials were trials in which a perturbation occurred, and half were trials in which one did not. The trials were grouped into 8 blocks each with 5 perturbation and 5 non-perturbation trials in a random order, thus leading to 40 perturbation and 40 non-perturbation trials total. This was repeated for both ankles and the DP and IE directions.

#### 3) Data Analysis for Impedance Experiments

For both standing and walking experiments, non-rejected trials were averaged. The differential torque and differential position were found starting at the onset of perturbation over a 100 ms window. From differential position, a five-point midpoint numerical differentiation was used to find the differential velocity and differential acceleration. A $2^{nd}$ order linear, time-invariant model was assumed for the impedance model and constrained linear regression analysis was used to find the stiffness, damping, and inertial estimates.

The quality of the fit was found by the percentage of variance accounted for (%VAF) [16] by the model when compared to the actual differential torque measurement. All position and torque curves were shifted along the x-axis such that zero in time represented the perturbation onset. The level of symmetry between the non-dominant and dominant limbs was determined as the ratio of the non-dominant stiffness to the dominant stiffness.

For the standing experiments, no trials were discarded, as the torque and perturbation curves were highly consistent across trials. For the walking experiments, however, greater care had to be taken due to the inherit variability in human walking data. Trials were excluded based on foot placement (−0.5 cm/+2.5 cm from the DP rotation axis and ±1.0 cm from IE rotation axis), and if either the torque or position trajectories were further than 3 standard deviations from the mean. Non-perturbation trial trajectories were averaged together. The position and torque trajectories from trials with perturbations had the respective averaged non-perturbation trajectory subtracted from them with each perturbation trial trajectory shifted along the y-axis such that the difference at perturbation onset was zero. The differential torque and position trajectories were then averaged together before being fit to the impedance model.

#### 4) Results of Ankle Impedance Experiments

The results of the standing and walking quantification for both non-dominant and dominant ankles and the IE and DP directions are highly reliable (Table 3), evidenced by the high %VAF across all the subjects and experimental conditions. The mean %VAF was higher than 95.5% for all 8 measurement conditions, i.e., non-dominant/dominant, DP/IE, and standing/walking conditions.

**TABLE 3:** Standing and Walking Impedance Quantification Results, Standard Deviations Are Shown In The Parentheses

	STANDING						WALKING					
	Non-dom. Stiffness (Nm/rad)	Non-dom. % VAF	Dom. Stiffness (Nm/rad)	Dom. % VAF	Symmetry	Symmetry Range	Non-dom. Stiffness (Nm/rad)	Non-dom. % VAF	Dom. Stiffness (Nm/rad)	Dom. % VAF	Symmetry	Symmetry Range
DP	207.3 (79.8)	99.4 (0.2)	240.1 (57.0)	99.6 (0.1)	0.87 (0.30)	0.60 - 1.34	403.5 (152.9)	99.3 (0.6)	359.9 (128.5)	99.2 (0.2)	1.11 (0.29)	0.76 - 1.48
IE	19.9 (3.8)	97.8 (0.4)	20.0 (8.1)	98.4 (1.1)	1.10 (0.37)	0.78 - 1.71	18.5 (8.2)	95.5 (3.6)	17.3 (7.4)	96.6 (3.2)	1.30 (0.84)	0.52– 2.72

Only stiffness results were presented since it has been shown in previous research that the stiffness component is the main contributor to the torque about the ankle that is induced by the perturbation [14], [15], [16]. As expected, for both standing and walking, the stiffness in the DP direction was higher than that in the IE [12], [14]. The average symmetry values may give the impression that the non-dominant ankle consistently has a lower stiffness than the dominant limb. However, a wide range of symmetry across subjects indicates that the symmetry between dominant versus non-dominant ankles is highly subject-specific with no obvious trend in this sample of five subjects. These results support the conclusion that our system is sensitive enough to detect differences in bilateral ankle impedance in 2-DOF during both standing and walking.

## IV. Discussion

Prior to the development of our new system, existing works to assess postural balance were limited to single force plate platforms [21], [22], while those to measure impedance were limited by single control modes and a limited range of perturbation motion [24], [25]. In this study, we introduced the twin dual-axis platform system which is capable of bilateral measurement of postural balance while simulating various environmental conditions and ankle impedance characterization in 2-DOF during both standing and walking.

With these capabilities, our presented system can be used to expand on previous studies to improve quantification of lower limb biomechanics by providing bilateral information of ankle impedance and/or postural balance control under diverse environmental conditions.

If our system is applied in populations with motor disabilities (e.g., stroke, cerebral palsy), the biomechanical information recorded may be used to develop new robotics that restore lost ability. For example, information on human postural sway may inform the development of balance controllers used in assistive exoskeletons or robot-assisted balance training programs that aim to improve postural balance control [3], [7]. Moreover, the comprehensive ankle impedance information generated by our system would allow for safer, and more effective coupled human-robot systems, which may alter their mechanical properties in real-time to improve force translation during gait or other movements [17], [40], [41].

To demonstrate the capabilities of this platform system, we designed four distinct human experiments. The first two experiments quantified postural balance in compliant impedance-controlled environments with various stiffnesses, and a sinusoidal perturbation setting with various frequencies and amplitudes. The second two experiments quantified bilateral ankle impedance in 2-DOF during standing and walking. The results of these experiments support the conclusion that our platform has the sensitivity to detect differences in standing postural balance under difference environmental conditions and bilateral differences in ankle impedance during standing and walking.

The capabilities of this system extend beyond the modalities demonstrated in this work. With control modes of velocity, position, and torque, a variety of previously unimplemented mechanical environments may be simulated to elucidate new information on the dynamics of ankle impedance, postural balance, and its rehabilitation. Future studies will be conducted to quantify postural balance and ankle impedance in disabled populations including individuals with stroke or multiple sclerosis.

## References

[1] P. T. Alpert, “Postural balance,” Home Health Care Manage. Pract., vol. 25, no. 6, pp. 279–281, 2013.

[2] E. Saruco, “Anodal tDCS over the primary motor cortex improves motor imagery benefits on postural control: A pilot study,” Sci. Rep., vol. 7, no. 1, pp. 1–9, Mar. 2017. 28352100 10.1038/s41598-017-00509-w PMC5428691

[3] A. R. Emmens, E. H. F. van Asseldonk, and H. van der Kooij, “Effects of a powered ankle-foot orthosis on perturbed standing balance,” J. NeuroEng. Rehabil., vol. 15, no. 1, pp. 1–13, Dec. 2018. 29914505 10.1186/s12984-018-0393-8 PMC6006747

[4] J.-H. Park, S. Kim, M. A. Nussbaum, and D. Srinivasan, “Effects of two passive back-support exoskeletons on postural balance during quiet stance and functional limits of stability,” J. Electromyogr. Kinesiol., vol. 57, Apr. 2021, Art. no. 102516. 10.1016/j.jelekin.2021.102516 33493784

[5] J. A. Saglia, “Design and development of a novel core, balance and lower limb rehabilitation robot: Hunova,” in Proc. IEEE 16th Int. Conf. Rehabil. Robot. (ICORR), Jun. 2019, pp. 417–422. 10.1109/ICORR.2019.8779531 31374665

[6] E. Swinnen, D. Beckwée, R. Meeusen, J.-P. Baeyens, and E. Kerckhofs, “Does robot-assisted gait rehabilitation improve balance in stroke patients? A systematic review,” Topics Stroke Rehabil., vol. 21, no. 2, pp. 87–100, Mar. 2014. 10.1310/tsr2102-87 24710969

[7] L. Hennington, V. Nalam, M. C. Eikenberry, C. L. Kinney, and H. Lee, “Visuomotor ankle training on a stiffness-controlled robotic platform improves ankle motor control and lower extremity function in chronic stroke survivors,” IEEE Trans. Med. Robot. Bionics, vol. 1, no. 4, pp. 237–246, Nov. 2019.

[8] G. Morone, “Robot-assisted gait training for stroke patients: Current state of the art and perspectives of robotics,” Neuropsychiatric Disease Treatment, vol. 13, pp. 1303–1311, May 2017. 28553117 10.2147/NDT.S114102 PMC5440028

[9] D. Winter, “Human balance and posture control during standing and walking,” Gait Posture, vol. 3, no. 4, pp. 193–214, Dec. 1995.

[10] D. A. Winter, A. E. Patla, F. Prince, M. Ishac, and K. Gielo-Perczak, “Stiffness control of balance in quiet standing,” J. Neurophysiol., vol. 80, no. 3, pp. 1211–1221, Sep. 1998. 9744933 10.1152/jn.1998.80.3.1211

[11] M. J. Warnica, T. B. Weaver, S. D. Prentice, and A. C. Laing, “The influence of ankle muscle activation on postural sway during quiet stance,” Gait Posture, vol. 39, no. 4, pp. 1115–1121, Apr. 2014. 24613374 10.1016/j.gaitpost.2014.01.019

[12] H. Lee, E. J. Rouse, and H. I. Krebs, “Summary of human ankle mechanical impedance during walking,” IEEE J. Transl. Eng. Health Med., vol. 4, pp. 1–7, 2016. 10.1109/JTEHM.2016.2601613 PMC5067112 27766187

[13] D. A. Winter, “Energy generation and absorption at the ankle and knee during fast, natural, and slow cadences,” Clin. Orthopaedics, vol. 175, pp. 147–154, May 1983. 6839580

[14] V. Nalam, E. Adjei, and H. Lee, “Quantification and modeling of ankle stiffness during standing balance,” IEEE Trans. Biomed. Eng., vol. 68, no. 6, pp. 1828–1837, Jun. 2021. 32915720 10.1109/TBME.2020.3023328

[15] M. Casadio, P. G. Morasso, and V. Sanguineti, “Direct measurement of ankle stiffness during quiet standing: Implications for control modelling and clinical application,” Gait Posture, vol. 21, no. 4, pp. 410–424, Jun. 2005. 15886131 10.1016/j.gaitpost.2004.05.005

[16] H. Lee, H. I. Krebs, and N. Hogan, “Multivariable dynamic ankle mechanical impedance with relaxed muscles,” IEEE Trans. Neural Syst. Rehabil. Eng., vol. 22, no. 6, pp. 1104–1114, Nov. 2014. 24686292 10.1109/TNSRE.2014.2313838 PMC4696764

[17] L. Chen, C. Wang, X. Song, J. Wang, T. Zhang, and X. Li, “Dynamic trajectory adjustment of lower limb exoskeleton in swing phase based on impedance control strategy,” Proc. Inst. Mech. Eng., I, J. Syst. Control Eng., vol. 234, no. 10, pp. 1120–1132, Nov. 2020.

[18] W. Huo, S. Mohammed, Y. Amirat, and K. Kong, “Active impedance control of a lower limb exoskeleton to assist sit-to-stand movement,” in Proc. IEEE Int. Conf. Robot. Autom. (ICRA), May 2016, pp. 3530–3536.

[19] S.-H. Cho, M.-H. Choi, and B.-O. Goo, “Effect of smart phone use on dynamic postural balance,” J. Phys. Therapy Sci., vol. 26, no. 7, pp. 1013–1015, 2014. 10.1589/jpts.26.1013 PMC4135186 25140085

[20] E. Antoniadou, “Reliability and validity of the mCTSIB dynamic platform test to assess balance in a population of older women living in the community,” J. Musculoskeletal Neuronal Interact., vol. 20, no. 2, pp. 185–193, 2020. PMC7288384 32481234

[21] N. Nawayseh and M. J. Griffin, “Effect of frequency, magnitude and direction of translational and rotational oscillation on the postural stability of standing people,” J. Sound Vibrat., vol. 298, no. 3, pp. 725–754, Dec. 2006.

[22] D. Sun, Y. Gu, Q. Mei, Y. Shao, J. Sun, and J. Fernandez, “Effect of heel heights on female postural control during standing on a dynamic support surface with sinusoidal oscillations,” J. Motor Behav., vol. 49, no. 3, pp. 281–287, May 2017. 10.1080/00222895.2016.1191423 27588676

[23] A. Roy, “Measurement of human ankle stiffness using the anklebot,” in Proc. IEEE 10th Int. Conf. Rehabil. Robot., Jun. 2007, pp. 356–363.

[24] E. J. Rouse, L. J. Hargrove, E. J. Perreault, M. A. Peshkin, and T. A. Kuiken, “Development of a mechatronic platform and validation of methods for estimating ankle stiffness during the stance phase of walking,” J. Biomech. Eng., vol. 135, no. 8, pp. 1–8, Aug. 2013. 10.1115/1.4024286 PMC3705977 23719922

[25] E. M. Ficanha, G. A. Ribeiro, and M. Rastgaar, “Design and evaluation of a 2-DOF instrumented platform for estimation of the ankle mechanical impedance in the sagittal and frontal planes,” IEEE/ASME Trans. Mechatronics, vol. 21, no. 5, pp. 2531–2542, Oct. 2016.

[26] V. Nalam and H. Lee, “Development of a two-axis robotic platform for the characterization of two-dimensional ankle mechanics,” IEEE/ASME Trans. Mechatronics, vol. 24, no. 2, pp. 459–470, Apr. 2019.

[27] V. Phan, “Standing postural balance under multi-directional perturbations,” in Proc. 45th Annu. Meeting Amer. Soc. Biomechanics (ASB), Aug. 2021, p. 143.

[28] V. Phan and H. Lee, “Prediction of compliant environments during postural balance using deep learning,” in Proc. 45th Annu. Meeting Amer. Soc. Biomech. (ASB), Aug. 2021, p. 142.

[29] J. Perry, Gait Analysis: Normal and Pathologic Functions. Thorofare, NJ, USA: Slack Inc., 1992.

[30] D. Ludvig and R. E. Kearney, “Real-time estimation of intrinsic and reflex stiffness,” IEEE Trans. Biomed. Eng., vol. 54, no. 10, pp. 1875–1884, Oct. 2007. 17926686 10.1109/TBME.2007.894737

[31] Cumulative Percent Distribution of Population by Height and Sex: 2007 to 2008, Cumulative Percent Distribution of Population by Weight and Sex: 2007 to 2008, Statistical Abstract. of the US, U.S. Census Bureau, Suitland, MD, USA, 2011, p. 135.

[32] B. Dasgupta and T. S. Mruthyunjaya, “The stewart platform manipulator: A review,” Mechanism Mach. Theory, vol. 35, no. 1, pp. 15–40, Jan. 2000.

[33] E. V. Zabre-Gonzalez, L. Riem, P. A. Voglewede, B. Silver-Thorn, S. R. Koehler-McNicholas, and S. A. Beardsley, “Continuous myoelectric prediction of future ankle angle and moment across ambulation conditions and their transitions,” Frontiers Neurosci., vol. 15, p. 986, Aug. 2021. 10.3389/fnins.2021.709422 PMC8416349 34483828

[34] M. Kouzaki and K. Masani, “Reduced postural sway during quiet standing by light touch is due to finger tactile feedback but not mechanical support,” Exp. Brain Res., vol. 188, no. 1, pp. 153–158, Jun. 2008. 18506433 10.1007/s00221-008-1426-5

[35] Kollmorgen. (2011). AKD: User Guide. [Online]. Available: https://www.kollmorgen.com/sites/default/files/akd/documentation/user%20guideAKD%20User%20Guide%20EN%20Rev%20J.pdf

[36] T. A. Stoffregen, K. Yoshida, S. Villard, L. Scibora, and B. G. Bardy, “Stance width influences postural stability and motion sickness,” Ecological Psychol., vol. 22, no. 3, pp. 169–191, Jul. 2010.

[37] B. Gear, Micron TRUE Planetary? Gearheads, document P-8741-BG, Oct. 2019.

[38] N. Hogan and S. P. Buerger, “Impedance and interaction control,” in Robotics and Automation Handbook. New York, NY, USA: CRC Press, 2005.

[39] R. Q. van der Linde and P. Lammertse, “HapticMaster—A generic force controlled robot for human interaction,” Ind. Robot, Int. J., vol. 30, no. 6, pp. 515–524, Dec. 2003.

[40] J. Chen, “A pediatric knee exoskeleton with real-time adaptive control for overground walking in ambulatory individuals with cerebral palsy,” Frontiers Robot. AI, vol. 8, p. 173, Jun. 2021. 10.3389/frobt.2021.702137 PMC8249803 34222356

[41] S. Hussain, S. Q. Xie, and P. K. Jamwal, “Adaptive impedance control of a robotic orthosis for gait rehabilitation,” IEEE Trans. Cybern., vol. 43, no. 3, pp. 1025–1034, Jun. 2013. 23193241 10.1109/TSMCB.2012.2222374

